# Excess all-cause mortality during the first wave of the COVID-19 epidemic in France, March to May 2020

**DOI:** 10.2807/1560-7917.ES.2020.25.34.2001485

**Published:** 2020-08-27

**Authors:** Anne Fouillet, Isabelle Pontais, Céline Caserio-Schönemann

**Affiliations:** 1 Santé publique France, Data science Division, Saint-Maurice, France

**Keywords:** all-cause, mortality, COVID-19, reactive surveillance, France

## Abstract

Through a weekly all-cause mortality surveillance system, we observed in France a major all-cause excess mortality from March to May 2020, concomitant with the coronavirus disease (COVID-19) epidemic. The excess mortality was 25,030 deaths, mainly among elderly people. Five metropolitan regions were the most affected, particularly Île-de-France and the Grand-Est regions. Assessing the excess mortality related to COVID-19 is complex because of the potential protective effect of the lockdown period on other causes of mortality.

Since 9 March 2020 (week 11), a significant excess mortality has been observed at national level in France, with a sharp increase in mortality from mid-March, reaching the highest level in week 14 and then decreasing. This dramatic increase in mortality was concomitant with the COVID-19 epidemic.

This paper aims to describe the temporal evolution of all-cause mortality during the COVID-19 epidemic in France and to provide an estimate of the excess all-cause mortality both at national and regional levels.

## A reactive all-cause mortality surveillance

Since 2005, the French public health agency (Santé Publique France) has been following up the all-cause mortality routinely, based on the administrative data transmitted by the French National Institute for Statistic and Economic Studies (INSEE). The mortality surveillance is based on ca 3,000 civil status offices of city halls dispatched all over the territory (including overseas) [1]. For each death, administrative information is collected (age, sex, date and place of death, date of registration). The medical causes of death are not recorded.

This sample from 3,000 cities represents 77% of the total French mortality, varying from 42% to 98% in the 100 districts [1]. In order to analyse mortality at the level of all cities, the number of deaths recorded from the sample in each district was divided by the coverage rate of the system at the district level. Numbers of deaths estimated at district level were aggregated to obtain estimated numbers of deaths at regional and national levels.

We obtained the excess all-cause mortality from the difference between the estimated number of deaths and the expected number of deaths (baseline). The weekly expected number of deaths was provided by a statistical model developed by the European monitoring of excess mortality for public health action network (EuroMOMO, www.euromomo.eu) [2]. This common model is used weekly by 24 European countries or regions and uses a time-series Poisson regression to predict the baseline, adjusted for a linear or nonlinear trend and seasonal variation.

The excess deaths are expressed in proportion to the baseline (excess mortality × 100, divided by the baseline). A standardised indicator around the baseline (z-score) is also calculated, enabling standardised comparison of mortality variations at different geographical levels. The analysis is stratified by age groups (< 15, 15–44, 45–64, 65–84 and ≥ 85 years) and sex at national and regional levels.

The use of mortality data in the frame of public health surveillance and epidemiological studies has been authorised by the French National Commission for Data Protection and the Liberties (CNIL).

## Estimates of excess all-cause mortality

**Figure 1:**
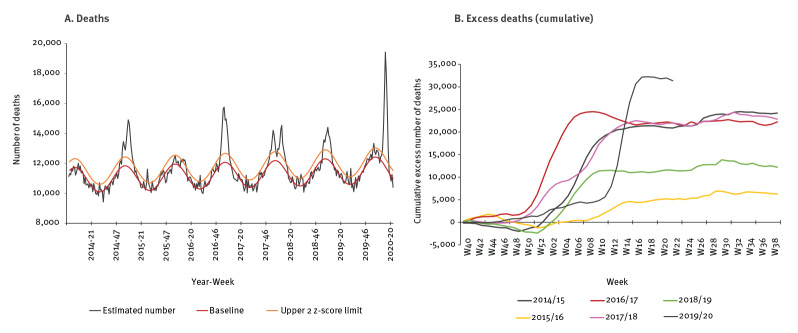
Overall and cumulative excess all-cause and all-age mortality, France, calendar week 40, 2013−22, 2020

From week 1 to week 10 in 2020, the estimated number of deaths varied around the expected value within 2 z-scores. The study period covered 2 March to 31 May 2020 (calendar weeks 10–22). Mortality increased sharply in week 11 in 2020 (crossing 2 z-scores) until week 14 in 2020. In week 14, the estimated number of deaths was 7,100 deaths (+60%) above the expected number of deaths (n = 11,800 deaths) (Figure 1). The mortality then decreased until week 18. The estimated number of deaths returned to the expected range from week 18 to week 22 (Figure 1). Overall for the study period, the estimated number of deaths was 16.6% above the baseline for the whole of France (n = 25,030 excess deaths) (Table).

**Table:** Estimated number of all-cause deaths, excess mortality, proportion among the expected number of deaths and z-score, by age group and by region, France, 2 March−31 May 2020 (n = 175,801)

	15–64 years-old				Older than 65 years				All ages			
	Estimated number of deaths	Excess	%	z-score	Estimated number of deaths	Excess	%	z-sore	Estimated number of deaths	Excess	%	z-score
France	**23,191**	**+1,513**	**+7.0^a^**	**8.6**	**151,652**	**+23,400**	**+18.2^a^**	**25.2**	**175,801**	**+25,027**	**+16.6^a^**	**25.4**
Auvergne-Rhône-Alpes	2,201	−11	−0.5	−0.3	**17,271**	**+2,550**	**+17.3^a^**	**12**	**19,579**	**+2,559**	**+15.0^a^**	**11.2**
Bourgogne-Franche-Comté	985	+42	+4.5	1.1	**7,807**	**+1,376**	**+21.4^a^**	**13.1**	**8,820**	**+1,421**	**+19.2^a^**	**13.9**
Britanny	1,154	+10	+0.9	0.2	**7,535**	**−353**	**−4.5^a^**	**−3.2**	**8,712**	**−329**	**−3.6^a^**	**−2.9**
Centre-Val de Loire	861	+21	+2.5	0.4	**6,239**	**+550**	**+9.7^a^**	**5.4**	**7,133**	**+570**	**+8.7^a^**	**5.4**
Corsica	100	+6	+6.4	−0.4	**717**	**+82**	**+12.9^a^**	**2.8**	**817**	**+88**	**+12.1^a^**	**2.6**
Grand Est	2,004	+82	+4.3	1.6	**15,785**	**+4,709**	**+42.5^a^**	**27.6**	**17,855**	**+4,812**	**+36.9^a^**	**27**
Hauts-de-France	2,481	+110	+4.6	1.7	**13,592**	**+2,432**	**+21.8^a^**	**12.9**	**16,152**	**+2,555**	**+18.8^a^**	**12**
Île-de-France	**4,515**	**+1,390**	**+44.5^a^**	**20.4**	**26,230**	**+10,659**	**+68.5^a^**	**46.3**	**30,979**	**+12,052**	**+63.7^a^**	**46.9**
Normandy	1,248	+67	+5.7	1.8	**7,637**	**+554**	**+7.8^a^**	**5.6**	**8,928**	**+640**	**+7.7^a^**	**5.6**
Nouvelle-Aquitaine	2,062	−92	−4.3	−2	**13,741**	**−549**	**−3.8^a^**	**−3.2**	**15,868**	**−634**	**−3.8^a^**	**−3.5**
Occitanie	1,981	+20	+1.0	0.2	12,673	+249	+2.0	1.4	14,731	+285	+2.0	1.5
Provence-Alpes-Côte d’Azur	1,568	−52	−3.2	−1.2	**11,758**	**+752**	**+6.8^a^**	**5**	**13,382**	**+697**	**+5.5^a^**	**4.2**
Pays de la Loire	1,239	−68	−5.2	−1.8	**8,050**	**+307**	**+4.0^a^**	**2.2**	9,338	+240	+2.6	1.6
Réunion Island	305	−33	−9.8	−1.9	899	−60	−6.3	−1.8	**1,235**	**−91**	**−6.9^a^**	**−2.2**
Mayotte	**100**	**+24**	**+31.6^a^**	**2.2**	**142**	**+58**	**+70.7^a^**	**5.5**	**268**	**+83**	**+44.9^a^**	**4.6**
Guadeloupe	176	+24	+15.8	1.7	710	+33	+4.9	1	896	+63	+7.6	1.7
Martinique	138	-6	−4.2	−0.8	**758**	**+69**	**+10.0^a^**	**2.3**	905	+62	+7.4	1.8
French Guiana	73	−21	**−22.3^a^**	−2.3	110	−18	−14.1	−1.5	**203**	**−46**	**−18.5^a^**	**−2.7**

^a^ The estimated number of deaths is significantly above or below the expected number of deaths (numbers in bold).

The cumulative excess mortality from week 40 in 2019 to week 22 in 2020 (30 September 2019–31 May 2020) exceeded cumulative excess mortality of the five full previous periods (from week 40 to 39 of the next year) (Figure 1).

**Figure 2:**
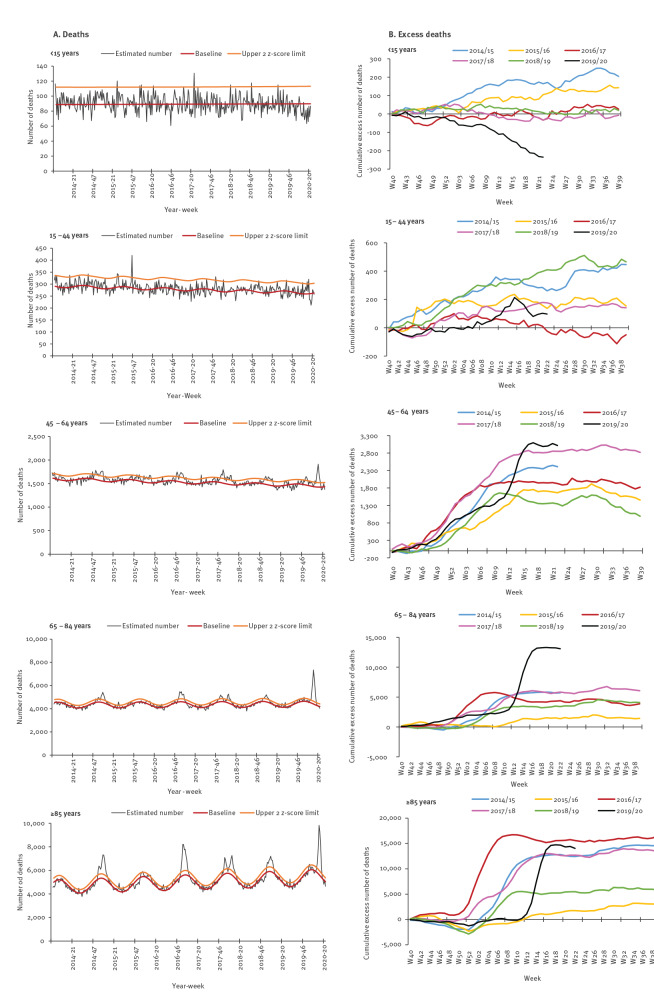
Overall and cumulative excess all-cause mortality by age group, France, calendar week 40 in 2013 to week 22 in 2020

**Figure 3:**
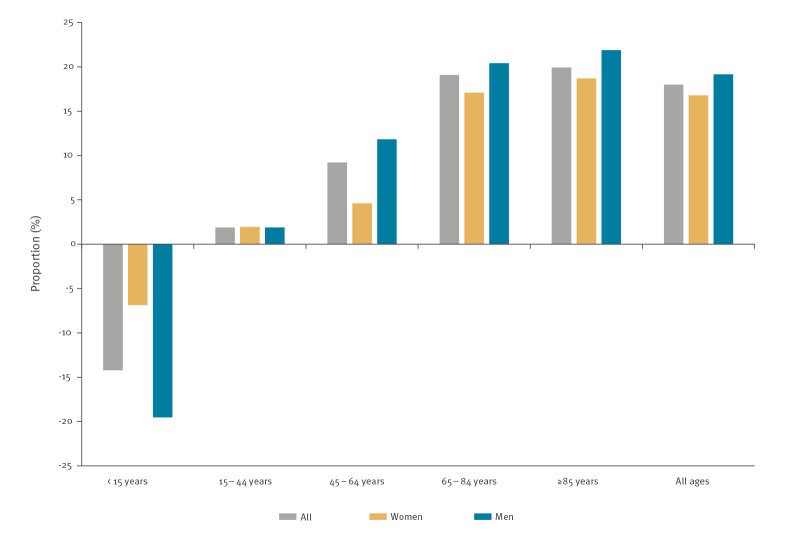
Proportion of excess all-cause mortality among the expected number of deaths, by age group and sex, France, 2 March−31 May 2020 (n = 175,801)

Elderly people (≥ 65 years) were the most affected (Figure 2). From week 10 to week 22 in 2020, 23,400 excess deaths were estimated in this age group (+18.2%), while persons aged 15–64 years experienced a moderate excess of deaths (+1,510 deaths, +7% compared with baseline) (Table). In this age group, the most affected were also the oldest (45–64 years) and more specifically men (+12% vs +5% in women compared with the baseline) (Figures 2 and 3). The estimated number of deaths in persons aged 15–44 years was comparable to the expected number, for both men and women. In elderly people, the proportion of all-cause excess deaths for men (19.1%) was greater than for women (16.7%). The estimated number of deaths in children (< 15 years-old) was lower than the expected value (−14%, n = −170 deaths), particularly for boys (−20%).

In comparison with the five full previous seasons, the cumulative all-cause excess mortality was close to three other seasons (2014/15, 2016/17 and 2017/18) for adults older than 85 years and to season 2017/18 for adults aged 45–64 years, but was much greater for 65–84 year-old (Figure 2).

## Major geographical disparities of excess mortality

Five metropolitan regions were particularly affected, with very high to exceptional excess mortality (z-scores value: 11–46.7) (Table). Île-de-France (Paris area) was the most impacted region with +12,050 deaths in excess (+63.7% compared with the baseline), followed by the region Grand-Est (+4,810 deaths, +36.9%) (Table). Excess mortality in the regions Auvergne-Rhône-Alpes, Bourgogne-Franche-Comté and Hauts-de-France ranged from +15% to +20%.

Five other regions experienced a moderate to elevated excess all-cause mortality with z-scores varying from 2 to 5.4. Eight regions had no observed excess mortality. Among these last regions, the estimated number of deaths was significantly lower than the baseline (ca −4%) in two regions (Nouvelle-Aquitaine and Brittany).

**Figure 4:**
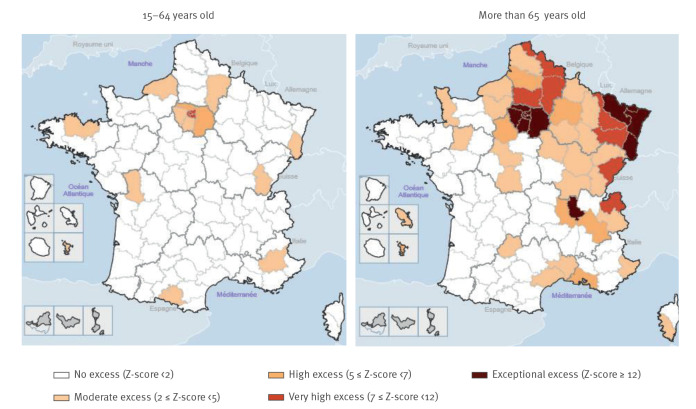
Level of all-cause excess deaths by county and age, France, 2 March−31 May 2020 (n = 175,801)

Spatial disparities were noticed within the regions, with 12 districts experiencing an exceptional excess mortality, including all Île-de-France districts (Figure 4). Excess mortality among adults aged 15–64 years was predominantly observed in Île-de-France.

## Discussion

In the absence of medical information in civil status data, it cannot be estimated which part of the all-cause excess mortality is attributable to COVID-19. During this unusual pandemic, two complementary surveillance systems were implemented to report COVID-19 deaths in hospitals and long-term care facilities in France [3], but no system was able to report the COVID-19 mortality at home. A total of 29,200 COVID-19 deaths were reported by these two systems during the study period [4]. This number of COVID-19-related deaths may be overestimated, since virological testing (PCR) was not systematically performed for all deaths reported by long-term care facilities.

The difference between our estimate of the all-cause excess mortality and the reported number of COVID-19 deaths in hospitals and long-term care facilities reflects the complexity to precisely estimate the epidemic impact since COVID-19 induced:

- an increase in deaths directly associated to severe acute respiratory disease coronavirus 2 (SARS-CoV-2),
- an increase in deaths due to other severe health conditions left untreated because of the impact of the epidemic on the health system and the lockdown,
- a decrease in mortality due to the suspension of business, personal and tourism trips and activities during the lockdown period,
- a decrease in mortality following an earlier death of the most frail persons who could have died from COVID-19 several days or weeks before they would have died without the epidemic.

The baseline corresponds to a mortality situation in absence of events that influence mortality. In the epidemic context, the lockdown period may have a protective effect on mortality and the number of deaths during the epidemic may be lower than the calculated baseline. The protective effect may concern external causes of deaths (such as traffic or occupational accidents), deaths related to air pollution or to other communicable diseases. This hypothesis is consistent with the situation observed in two regions with a lower mortality than expected that started 3 weeks after the beginning of the lockdown and remained until the end of the lockdown period and even longer in one of the two regions. Such results were also reported in Portugal, for persons younger than 55 years [5]. Further analysis using medical causes of deaths will allow disentangling the impact of lockdown on severe illnesses.

Different effects causing increases and decreases in mortality occur simultaneously during the same period. Providing an estimate for each effect is a great challenge. All-cause mortality data are insufficient to separate COVID-19-related deaths (directly or indirectly associated with the epidemic) from deaths related to other causes, but do provide rapid and valid information on excess mortality impact.

Spatial disparities in the excess mortality were mainly related to the spatial spread of the epidemic (data not shown) [4]. Further studies will be required to examine other factors that could explain disparities, in particular in Île-de-France where an excess mortality in 15–64-year-old adults was observed. Those multifactorial effects are mainly related to health status and socio-demographic and economic characteristics of the population residing in these territories, as well as medical and to do with health care geography. 

Our results by age group were consistent with previous studies that have reported a major impact on the elderly [5-8], but they differ for children. For children, our study showed a significantly smaller number of deaths than the baseline, whereas at the European level and in England, the number of deaths during the epidemic remained comparable to the baseline [6,7]. The estimate of excess mortality by sex is rarely explored. In Italy, the excess among men was also higher than among women [8].

Overall, excess all-cause mortality is an essential indicator of disease impact, particularly during unusual events such as the current pandemic. All-cause mortality monitoring contributes to guide public health decisions and actions in order to reduce its impact at national and local level. The French surveillance system also contributes to the excess mortality surveillance at the European level through the EuroMOMO network that is particularly of interest in case of international threats [7].
